# Dietary polyphenols and risk of breast cancer in a predominantly low-income population: a prospective analysis in the Southern Community Cohort Study (SCCS)

**DOI:** 10.1016/j.ajcnut.2025.03.017

**Published:** 2025-03-22

**Authors:** Lei Fan, Landon T Fike, Heather Munro, Danxia Yu, Hongwei Si, Martha J Shrubsole, Qi Dai

**Affiliations:** 1 Department of Medicine, Division of Epidemiology, Vanderbilt Epidemiology Center, Vanderbilt University Medical Center, Nashville, TN, United States; 2 Department of Radiology, Vanderbilt University Medical Center, Nashville, TN, United States; 3 Department of Medicine, Division of Epidemiology, International Epidemiology Field Station, Vanderbilt University Medical Center, Nashville, TN, United States; 4 Department of Food and Animal Sciences, Tennessee State University, Nashville, TN, United States

**Keywords:** polyphenols, phenolic acids, breast cancer, Southern Community Cohort Study, estrogen receptor/progesterone receptor, menopausal status

## Abstract

**Background:**

Few studies have examined the associations of specific dietary polyphenols with breast cancer (BC) risks or among non-Hispanic Black (NHB) female individuals in the United States.

**Objectives:**

We aim to evaluate the associations between total and subclasses of polyphenol intake and BC risk, stratified by body mass index (BMI), estrogen receptor (ER)/progesterone receptor (PR) status, menopausal status, and racial and ethnic subgroups.

**Methods:**

The study included 42,260 female participants from the Southern Community Cohort Study, a large prospective cohort of predominantly low-income NHB Americans. The dietary polyphenol components were assessed using a validated culturally sensitive 89-item food frequency questionnaire designed specifically for nutrient intakes in the South. Cox proportional hazards model was used to assess the associations after adjustment for confounders including sociodemographic and lifestyle factors.

**Results:**

Intakes of total polyphenols were higher in non-Hispanic white (1122 ± 727 mg/d) than in NHB female individuals (535±349 mg/d). Intakes of total polyphenol, particularly phenolic acids, were associated with reduced risk of BC incidence among female individuals with the ER+ and PR+ BC type comparing the highest to the lowest quintile [hazard ratio (HR) 0.69; 95% confidence interval (CI): 0.51, 0.94; *P*_-trend_ = 0.003; HR 0.70; 95% CI: 0.53, 0.95; *P*_-trend_ = 0.005, respectively]. Phenolic acid was inversely related to BC among postmenopausal female individuals (HR 0.76; 95% CI: 0.59, 0.97; *P*_-trend_ = 0.02) and female individuals with a BMI ≥ 25 kg/m^2^ (HR 0.77; 95% CI: 0.60, 0.98; *P*_-trend_ = 0.01) comparing the highest to the lowest quintile. Intakes of tyrosols were associated with increased risk of BC among NHB female individuals (HR 1.34; 95% CI: 1.03, 1.73; *P*_-trend_ = 0.01) and female individuals with a BMI ≥ 25 kg/m^2^ (HR 1.31; 95% CI: 1.04, 1.65; *P*_-trend_ = 0.004).

**Conclusions:**

In this predominantly low-income United States population, intakes of total polyphenol and phenolic acids were associated with reduced risk of BC among those with ER+ and PR+ BC type, postmenopausal, and female individuals with overweight/obesity.

## Introduction

Breast cancer (BC) is the most common neoplasia and the second leading cause of cancer death among American female individuals [1]. Although the death rate has dropped by 43% during 1989–2020, non-Hispanic Black (NHB) female individuals had the highest BC death rate (27.6 per 100,000) and the lowest 5-y survival for all molecular subtypes across all United States racial and ethnic subgroups [1,2]. Estrogen plays an essential role in breast carcinogenesis, particularly among postmenopausal female individuals [3]. Previous studies found that the risk of BC death among estrogen receptor (ER) and/or progesterone receptor (PR) positive patients, but not among those with triple-negative BC, was ≤4 times increased risk for NHB than for non-Hispanic White (NHW) patients [[4], [5], [6]].

Polyphenols represent a large group of over 10,000 natural bioactive compounds found in various fruits, vegetables, and beverages such as wine, tea, and coffee [7]. These compounds are components of a high-quality diet but are inadequately consumed by low-income individuals [8]. The major polyphenol classes include flavonoids, phenolic acids, stilbenes, and lignans. The structural variations in the polyphenol classes are responsible for their differing biological activities. Some polyphenols possess estrogenic or antiestrogenic effects by competing with estrogens to bind to ERs [[9], [10], [11], [12]] or inhibiting aromatases [13,14]. A number of prospective epidemiological studies have examined the associations between polyphenol exposures and the risk of BC. However, these studies have generated inconsistent results [11,[15], [16], [17], [18]]. Some studies showed that exposure to polyphenols was associated with a reduced risk of BC only among postmenopausal female individuals, female individuals with higher BMI, or for ER/PR-positive BC [10,17,19,20].

Previous studies examined the associations between the intakes of polyphenol-enriched food groups, including tea, coffee, fruits, and vegetables, and the risk of BC among NHB female individuals in the Black Women’s Health Study [21,22]. Although total vegetable consumption was associated with a decreased risk of ER-negative/PR-negative BC [21], consumption of tea, coffee, and caffeine was not associated with BC risk overall and according to menopausal status or hormone receptor status among NHB female individuals [22]. No study has examined the associations of total, classes, and major subclasses of polyphenol intake with risk of BC among primarily NHB female individuals. The Southern Community Cohort Study (SCCS) is a large prospective cohort study designed to study the root causes of cancer disparities and represents a predominantly low-income population at high risk of a suboptimal diet, of whom two-thirds identify as NHB. Investigating the polyphenol intakes and risk of BC incidence in the SCCS reveals whether dietary polyphenols contribute to a higher risk of BC, given that NHB females have the highest BC mortality. We aimed to evaluate the longitudinal associations between dietary intakes of total as well as classes, and major subclasses of polyphenols and the risk of BC. We further examined the associations stratified by BMI status, ER/PR status, menopausal status, and racial and ethnic subgroups.

## Methods

### Study population

**FIGURE 1:**
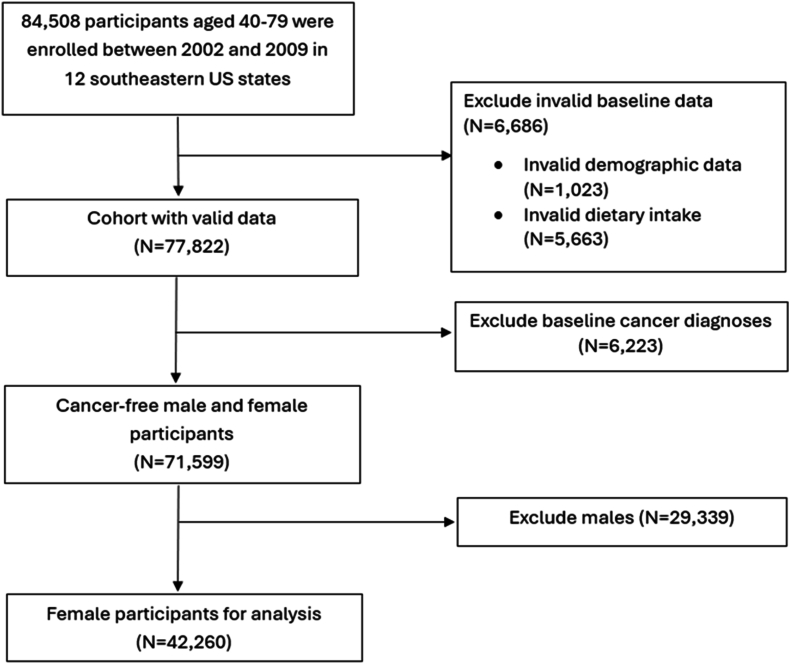
Flowchart of the Southern Community Cohort Study (SCCS) participants included in this study.

The SCCS is a prospective cohort study including roughly 85,000 adults aged 40–79 y at enrollment across 12 southeastern states. The SCCS was designed to address racial and socioeconomic cancer health disparities. The cohort methods are described in detail in a previous publication [23]. Briefly, participants were enrolled between March 2002 and September 2009 either in-person at community health centers (CHC, 86%) or by mail-based population sampling (14%). In this study, we excluded 6686 with invalid information on demographic data or dietary intakes, 6223 with baseline cancer diagnoses, and 29,339 males, leaving 42,260 female individuals included in this study (Figure 1). All participants provided written informed consent, and the study was approved by the Institutional Review Boards at Vanderbilt University Medical Center (registration number: 010345).

### Dietary assessment

The dietary components in the SCCS were assessed using a validated culturally sensitive 89-item food frequency questionnaire (FFQ) designed specifically for nutrient intakes of NHB Americans and non-Black Americans in the South [24], which was developed based on 24-h dietary recall data from the NHANES and the continuing Survey of Food Intakes [25]. Estimates of the amount of food in grams per day were calculated for each FFQ food item, and the distribution of foods that contributed to the calculation for each FFQ item was obtained using race, sex, and geographical region-specific food intake [25]. The percent weights of each of these foods were calculated for each FFQ food item using USDA recipe databases that contain the percent weights of 1878 unique ingredients contributing to each food variable. Each ingredient was then linked to the USDA Expanded Flavonoid Database (v. 1.1), which contains data from the USDA Database for the Flavonoid Content of Selected Foods (v. 3.2) and USDA Database for the Isoflavone Content of Selected Foods (v. 2.1) [26]**.** Each ingredient was also merged with the USDA Database for the Proanthocyanidin Content of Selected Foods (v. 2), largely using a manual review to assign each ingredient, taking into account the percent weights of ingredients and retention factors applied in the Expanded Flavonoid Database [27]**.** Likewise, to obtain data on other polyphenol classes, the ingredients were manually assigned to foods within Phenol-Explorer using standard recipes, ingredient lists, and patent information for certain ingredients [28]**.**

The detailed methods for calculating polyphenol intake for each participant have been previously reported [8]. In brief, USDA polyphenol data are presented in aglycone equivalents and account for food processing. Phenol-Explorer data are generally available as raw polyphenols. To combine the data from these 2 sources, Phenol-Explorer data were first converted to aglycone equivalents. Next, if the aglycone was already represented in the USDA data, as was the case for most flavonoids, the USDA values were used in place of Phenol-Explorer data as USDA polyphenol data are more likely to represent the foods consumed by United States participants. Some polyphenols in Phenol-Explorer have values obtained by multiple methods. Some foods contain polyphenols linked to the food matrix itself that are released after hydrolysis [29]. For these polyphenols, the values obtained by chromatography after hydrolysis were used. Next, retention factors were used when appropriate to account for changes during the cooking process. Finally, Phenol-Explorer and USDA data were merged to produce an SCCS-specific database for each demographic stratum and food variable. This allowed for the estimation of polyphenol intake for each participant.

Intake levels were assessed for total polyphenols as well as major classes and subclasses, including flavonoids [anthocyanins, chalcones, dihydrochalcones, dihydroflavonols, flavonols (oligomeric proanthocyanidins), flavanones, flavones, flavonols, and isoflavonoids], phenolic acids (hydroxybenzoic acids, hydroxycinnamic acids, hydroxyphenylacetic acids, and hydroxyphenylpropanoic acids), lignans, stilbenes alkylmethoxyphenols, alkylphenols, curcuminoids, furanocoumarins, hydroxybenzaldehydes, hydroxybenzoketones, hydroxycoumarins, hydroxyphenylpropenes, methoxyphenols, naphtoquinones, phenolic terpenes, and tyrosols. Several subclasses made a negligible contribution to polyphenol intake and would be less likely to have plausible biological effects given the low concentrations in the body. Thus, for this analysis, we focused on total polyphenols, major classes, and subclasses with a median intake in this cohort of 1 mg/d or greater [that is, flavanols (proanthocyanidins), flavonols, flavanones, anthocyanins, flavones, hydroxycinnamic acids, hydroxybenzoic acids, alkylphenols, alkylmethoxyphenols, and tyrosols] [8].

### Outcome ascertainment

Incident BC was obtained through linkage with the 12 SCCS state cancer registries and the National Death Index. Participants were followed from enrollment at baseline through the date of BC diagnosis, death, loss to follow-up, or 31 December, 2016, the date through which all cancer registries reported having complete data.

### Statistical analyses

Polyphenol intakes were log-transformed, adjusted for total energy intake using the residual energy adjustment described previously, and then back-transformed [30]. We adjusted for total energy intake because extraneous variation in nutrient intake resulting from variation in total energy intake that is unrelated to disease risk may weaken associations [30]. Energy-adjusted polyphenol intake was then grouped into quintiles based on the distribution within the whole cohort. Baseline characteristics of the cohort were compared across the quintiles of total polyphenols using Chi-square and analysis of variance tests. Hazard ratios (HRs) and 95% confidence intervals (CIs) were estimated using Cox proportional hazards models adjusted for age (continuous), race (NHB, NHW, and other), annual household income (<$15,000, $15,000–$25,000, $25,000–$50,000, >$50,000), enrollment source (CHC, general public), BMI (<25, 25–29.9, ≥30 kg/m^2^), physical activity [continuous, metabolic equivalent hours per day (MET-Hr/d)], smoking status and packyears (never smokers, former smokers, current <20 packyears, and current ≥20 packyears), alcohol drinking status (nondrinker, moderate drinker, more than moderate drinker), menopause status (premenopausal, postmenopausal), comorbidity index (continuous), total energy intake (continuous, kcal/d), overall diet quality [Healthy Eating Index (HEI)-2010, continuous], and family history of BC (no known family history, known family history). All racial and ethnic groups were self-reported by study participants in the SCCS. Participants were asked “Which of the following describes your racial or ethnic background?” Participants chose from the categories of White, Black/African-American, Hispanic/Latino, Asian or Pacific Islander, American Indian or Alaska Native, or other racial or ethnic group. The SCCS recruited participants from 2 enrollment sources: CHCs and the general population [23]. The CHCs are government-funded healthcare facilities that successfully recruited low-income individuals from rural and urban areas in the south, whereas the remainder was recruited from a stratified random sampling of the southern general population. The comorbidity index was derived from a modified Charlson Comorbidity Index and calculated for each participant by summing the number of self-reported diseases at baseline [31,32]. The HEI-2010 was a useful index of overall diet quality calculated by linking the FFQ data with MyPyramid Equivalents Database and scoring (0–100 points) based on the 2010 Dietary Guidelines for Americans [33,34]. The proportional hazards assumption was confirmed for the Cox models by plotting Schoenfield residuals against time and log time and analyzing for flat lines around 0. Tests for linear trends were assessed using the median value of each quintile as a continuous variable in the Cox models. First, we examined the association between intakes of total polyphenols and the risk of BC. Then, we investigated the associations between intakes of major classes of polyphenols and, in turn, subclasses of polyphenols and the risk of BC. Restricted cubic splines were used to examine possible nonlinear relationships between intakes of total polyphenols, classes and subclasses of polyphenols, and risk of BC [35]. Multivariable-adjusted analyses with 3 knots were conducted within the values between the first and 99th percentile to minimize the impact of potential outliers. Three knots were located at the 10th, 50th, and 90th percentiles of intakes of total polyphenols, classes, and subclasses of polyphenols. Stratified or heterogeneity analyses by hormonal receptor status of BC, menopausal status, race, and BMI were conducted to assess potential effect modifications for the polyphenols that were significantly associated with BC risk in the overall models. The significance of multiplicative interactions was evaluated by adding a corresponding interaction term in the models. Multiplicative interaction was evaluated using the likelihood ratio test. The records for individuals with missingness in covariates were not included in respective models. Sensitivity analysis was also conducted by excluding those who were followed up <12 mo after baseline. The data analyses used Statistical Analysis Software, version 9.4 (SAS Institute Inc.) and restricted cubic splines were plotted using R 4.2.0 (https://cran.r-project.org/). All *P* values are 2-sided and statistical significance was determined using an alpha level of 0.05.

## Results

Table 1 shows the baseline characteristics of individuals by quintile of total polyphenol intakes. This study included 42,260 female participants, with 28,082 (66.4%) NHB female individuals. The mean (SD) age at baseline was 52.2 (8.8) y. The FFQ-derived residual-model energy-adjusted mean (SD) intakes of total polyphenols were 723 (568) mg/d for all participants, 535 (349) mg/d for NHB female individuals, and 1122 (727) mg/d for NHW female individuals. In Supplemental Table 1, we presented the median intakes for all polyphenols (classes and subclasses) among female participants in the SCCS. In comparison to those with the lowest intake, individuals with higher intake were more likely to be recruited from the general population (17.4% compared with 7.4%), older [mean (SD) age, 53.9 (9.0) compared with 49.9 (7.9)], NHW (65.8% compared with 15.1%), postmenopausal (74.8% compared with 58.0%), physically active [mean (SD) MET-Hr, 21.4 (15.9) compared with 20.4 (15.9)], former or current smokers (62.3% compared with 50.5%), moderate drinkers (37.3% compared with 32.3%), and to have higher income (≥50,000 USD, 13.8% compared with 4.0%), a lower BMI (% of BMI <25, 25.5% compared with 16.6%), known family history of BC (11.1% compared with 9.2%) and a healthier overall diet [mean (SD) HEI, 60.9 (12.8) compared with 53.5 (10.6)]. On the basis of our previous report in the SCCS [8], the main sources of total polyphenols are regular coffee (31%), tea (20%), decaf coffee (7%), fruit juice (5%), apples and pears (4%), grapes and berries (3%), citrus juice (3%), high fiber cereal (3%), and beans (2%).

**TABLE 1:** Baseline characteristics of participants according to quintiles of energy-adjusted total polyphenol intake among female individuals in the Southern Community Cohort Study (*n* = 42,260).

	Quintile 1 (Low)	Quintile 2	Quintile 3	Quintile 4	Quintile 5 (high)
Sample size (*n*)	8452	8452	8452	8452	8452
Polyphenol range (mg/d)	<281	281–464	464–671	671–1070	1070–5120
Age (SD)	49.9 (7.9)	50.9 (8.3)	52.6 (8.9)	54.0 (9.2)	53.9 (9.0)
Race (%)					
Non-Hispanic Black	6953 (82.3)	7074 (83.7)	6723 (79.5)	5041 (59.6)	2291 (27.1)
Non-Hispanic white	1280 (15.1)	1133 (13.4)	1390 (16.4)	2939 (34.8)	5562 (65.8)
Other	202 (2.4)	213 (2.5)	306 (3.6)	444 (5.3)	550 (6.5)
Unknown (%)	17 (0.2)	32 (0.4)	33 (0.4)	28 (0.3)	49 (0.6)
Menopausal status					
Premenopausal (%)	3533 (41.8)	3129 (37.0)	2671 (31.6)	2303 (27.2)	2120 (25.1)
Postmenopausal (%)	4905 (58.0)	5307 (62.8)	5771 (68.3)	6137 (72.6)	6319 (74.8)
Unknown (%)	14 (0.2)	16 (0.2)	10 (0.1)	12 (0.1)	13 (0.2)
Annual household income (%)					
<$15,000	5296 (62.7)	4893 (57.9)	4615 (54.6)	4374 (51.8)	3952 (46.8)
$15,000–$25,000	1819 (21.5)	1975 (23.4)	1924 (22.8)	1798 (21.3)	1749 (20.7)
$25,000–$50,000	884 (10.5)	1037 (12.3)	1241 (14.7)	1295 (15.3)	1445 (17.1)
>$50,000	335 (4.0)	457 (5.4)	557 (6.6)	850 (10.1)	1168 (13.8)
Unknown (%)	118 (1.4)	90 (1.1)	115 (1.4)	135 (1.6)	138 (1.6)
Enrollment source (%)					
Community health center	7826 (92.6)	7691 (91.0)	7625 (90.2)	7412 (87.7)	6980 (82.6)
General population	626 (7.4)	761 (9.0)	827 (9.8)	1040 (12.3)	1472 (17.4)
BMI kg/m^2^ (%)					
<25	1403 (16.6)	1561 (18.5)	1462 (17.3)	1654 (19.6)	2152 (25.5)
25–29.9	2033 (24.0)	2055 (24.3)	2113 (25.0)	2222 (26.3)	2291 (27.1)
≥30	4864 (57.6)	4713 (55.8)	4764 (56.4)	4444 (52.6)	3904 (46.2)
Unknown (%)	152 (1.8)	123 (1.5)	113 (1.3)	132 (1.6)	105 (1.2)
Physical activity, MET-Hr/d (SD)	20.4 (15.9)	21.5 (16.3)	21.1 (15.8)	20.9 (15.7)	21.4 (15.9)
Unknown (%)	128 (1.5)	142 (1.7)	125 (1.5)	132 (1.6)	156 (1.8)
Smoking status (%)					
Never smoker	4067 (48.1)	4135 (48.9)	4023 (47.6)	3813 (45.1)	3014 (35.7)
Former smoker	1460 (17.3)	1589 (18.8)	1789 (21.2)	2071 (24.5)	2087 (24.7)
Current, <20 packyears	1857 (22.0)	1751 (20.7)	1629 (19.3)	1368 (16.2)	1329 (15.7)
Current, >20 packyears	1008 (11.9)	908 (10.7)	945 (11.2)	1128 (13.3)	1942 (23.0)
Unknown (%)	60 (0.7)	69 (0.8)	66 (0.8)	72 (0.9)	80 (0.9)
Alcohol intake (%)					
Nondrinker	4632 (54.8)	4563 (54.0)	4804 (56.8)	4772 (56.5)	4485 (53.1)
Moderate drinker	2730 (32.3)	2617 (31.0)	2704 (32.0)	2813 (33.3)	3149 (37.3)
More than moderate	1045 (12.4)	1209 (14.3)	894 (10.6)	799 (9.4)	741 (8.8)
Unknown (%)	45 (0.5)	63 (0.7)	50 (0.6)	68 (0.8)	77 (0.9)
Comorbidity index (SD)	1.9 (1.4)	1.9 (1.4)	1.9 (1.3)	1.9 (1.4)	1.9 (1.4)
Unknown (%)	135 (1.6)	153(1.8)	158 (1.9)	167 (2.0)	215 (2.5)
Total energy intake, kcal/d (SD)	1971 (1340)	2505 (1450)	2374 (1289)	2266 (1154)	1996 (871)
Healthy Eating Index (SD)	53.5 (10.6)	59.2 (11.0)	60.9 (11.5)	62.9 (12.0)	60.9 (12.8)
Family history of BC (%)					
No known family history	7674 (90.8)	7643 (90.4)	7627 (90.2)	7522 (89.0)	7516 (88.9)
Known family history	778 (9.2)	809 (9.6)	825 (9.8)	930 (11.0)	936 (11.1)

Note: *P* value obtained using Chi-square tests for categorical variables and ANOVA tests for continuous variables.

Abbreviations: ANOVA, analysis of variance; BC, breast cancer; SCCS, Southern Community Cohort Study; MET-Hr/d, metabolic equivalent hours per day.

### Total polyphenols, polyphenol classes and subclasses and BC

**FIGURE 2:**
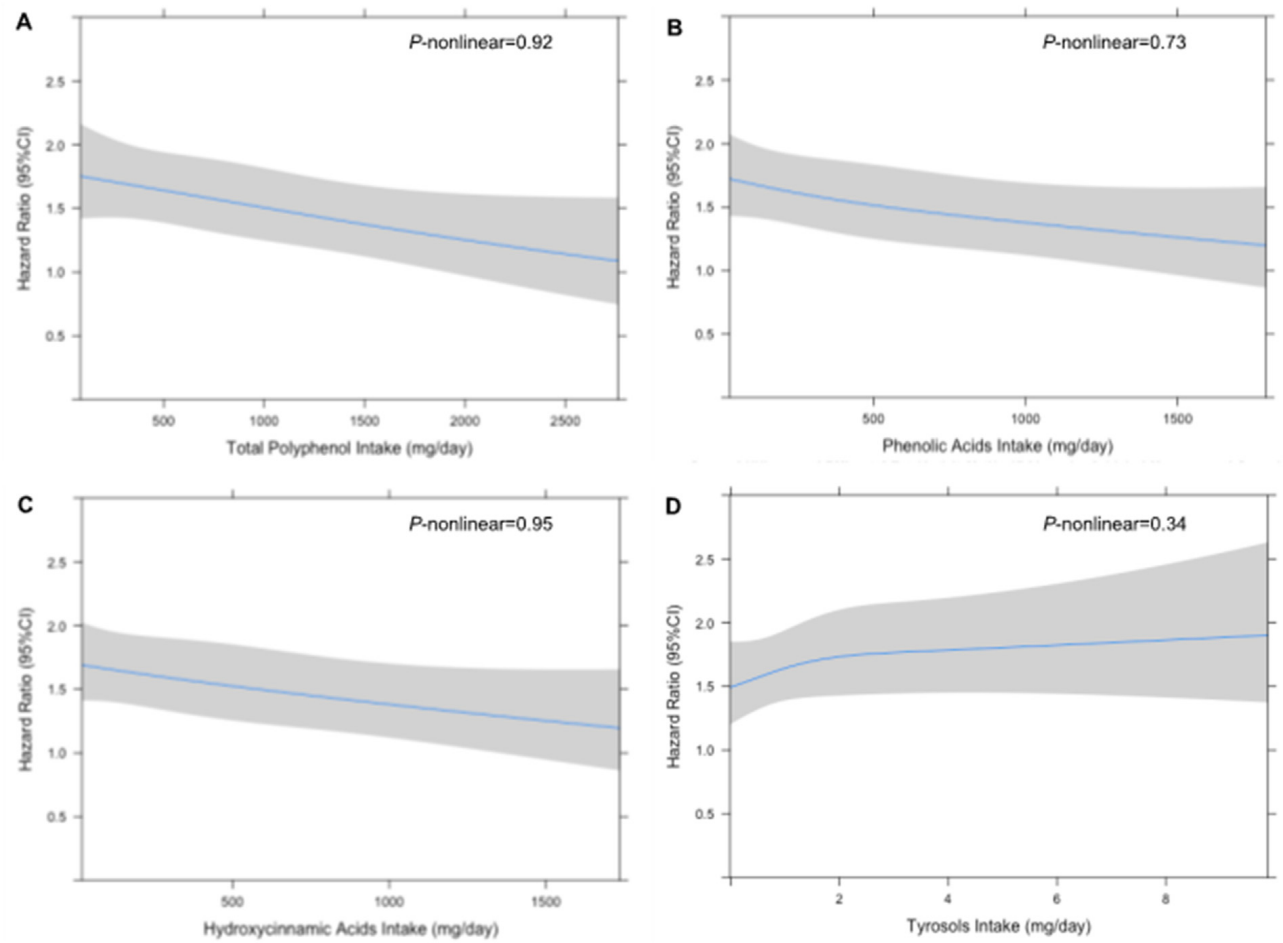
Multivariable-adjusted hazard ratios (HRs) and 95% confidence intervals (CIs) for associations between energy-adjusted polyphenol intake and breast cancer risk using restricted cubic splines analysis (*n* = 42,260). (A) total polyphenol, (B) phenolic acids, (C) hydroxycinnamic acids, and (D) tyrosols.

A total of 1141 incident BC cases were identified over a median follow-up of 11.2 y. There was some evidence of a trend toward an inverse association between total polyphenol intake and BC risk in the multivariable-adjusted model (HR 0.86; 95% CI: 0.69, 1.07; *P*_-trend_ < 0.05) comparing the highest to the lowest quintile (Table 2). Similarly, there was an inverse dose–response trend between phenolic acid intake and BC risk in multivariable-adjusted model (HR 0.82; 95% CI: 0.66, 1.02; *P*_-trend_ = 0.03) comparing the highest to the lowest quintile. Specifically, among polyphenol subclasses, there was evidence of a trend toward an inverse association between intakes of hydroxycinnamic acids and risk of BC (HR 0.87; 95% CI: 0.70, 1.08; *P*_-trend_ = 0.03). In contrast, intakes of tyrosols were significantly associated with increased risk of BC (HR 1.24; 95% CI: 1.00, 1.54; *P*_-trend_ = 0.01). All other classes and subclasses of polyphenols were not associated with the risk of BC. Restricted cubic spline analyses did not observe significant nonlinear associations between intakes of total polyphenols, phenolic acids, hydroxycinnamic acids, or tyrosols and the risk of BC (Figure 2).

**TABLE 2:** Associations between polyphenol intakes and breast cancer risk in the Southern Community Cohort Study (*n* = 42,260).

Polyphenol	Quintile 1 (lowest)		Quintile 2		Quintile 3		Quintile 4		Quintile 5 (highest)		*P*_-trend_
	HR	Cases	HR (95% CI)	Cases	HR (95% CI)	Cases	HR (95% CI)	Cases	HR (95% CI)	Cases	
Total polyphenols	1.00 (ref)	214	1.12 (0.92, 1.35)	249	0.97 (0.80, 1.18)	228	0.97 (0.79, 1.19)	248	0.86 (0.69, 1.07)	202	0.05
Flavonoids	1.00 (ref)	198	1.15 (0.95, 1.40)	244	1.02 (0.83, 1.26)	232	1.08 (0.88, 1.33)	247	1.00 (0.80, 1.23)	220	0.51
Flavanols	1.00 (ref)	206	1.06 (0.87, 1.29)	245	1.00 (0.82, 1.22)	245	0.93 (0.76, 1.14)	231	0.95 (0.77, 1.16)	214	0.35
Proanthocyanidins	1.00 (ref)	209	1.03 (0.85, 1.25)	223	1.10 (0.90, 1.33)	245	0.95 (0.78, 1.17)	221	0.95 (0.77, 1.18)	243	0.40
Flavonols	1.00 (ref)	199	1.14 (0.94, 1.39)	238	1.06 (0.86, 1.29)	229	1.15 (0.94, 1.41)	250	1.05 (0.85, 1.29)	225	0.91
Flavanones	1.00 (ref)	220	1.01 (0.84, 1.23)	230	0.92 (0.76, 1.12)	213	0.85 (0.70, 1.04)	217	0.94 (0.77, 1.15)	261	0.50
Anthocyanins	1.00 (ref)	190	1.11 (0.90, 1.35)	230	1.12 (0.91, 1.38)	244	1.10 (0.88, 1.36)	242	0.99 (0.78, 1.25)	235	0.41
Flavones	1.00 (ref)	174	1.33 (1.09, 1.63)	243	1.21 (0.98, 1.50)	234	1.21 (0.97, 1.50)	244	1.14 (0.91, 1.44)	246	0.99
Phenolic acids	1.00 (ref)	222	0.98 (0.80, 1.19)	225	1.08 (0.89, 1.31)	260	0.94 (0.78, 1.15)	231	0.82 (0.66, 1.02)	203	0.03
Hydroxycinnamic acids	1.00 (ref)	213	1.09 (0.90, 1.33)	244	1.08 (0.88, 1.32)	250	0.97 (0.80, 1.18)	229	0.87 (0.70, 1.08)	205	0.03
Hydroxybenzoic acids	1.00 (ref)	215	1.01 (0.83, 1.22)	235	1.01 (0.83, 1.23)	237	0.97 (0.80, 1.18)	224	0.95 (0.78, 1.17)	230	0.50
Lignans	1.00 (ref)	191	1.15 (0.94, 1.40)	236	1.14 (0.92, 1.40)	237	1.18 (0.94, 1.48)	257	1.05 (0.81, 1.36)	220	0.97
Stilbenes	1.00 (ref)	215	0.84 (0.68, 1.04)	200	0.99 (0.79, 1.24)	252	0.92 (0.73, 1.16)	235	0.95 (0.75, 1.20)	239	0.97
Other polyphenols											
Alkylphenols	1.00 (ref)	202	0.93 (0.76, 1.14)	190	1.07 (0.88, 1.31)	233	1.23 (1.01, 1.50)	270	1.07 (0.87, 1.33)	246	0.64
Alkylmethoxyphenols	1.00 (ref)	235	0.98 (0.81, 1.18)	219	1.12 (0.93, 1.36)	254	0.95 (0.79, 1.15)	222	0.89 (0.73, 1.10)	211	0.13
Tyrosols	1.00 (ref)	221	0.96 (0.79, 1.16)	222	0.99 (0.81, 1.21)	218	0.99 (0.82, 1.21)	219	1.24 (1.00, 1.54)	261	0.01

Models adjusted for age, race, household income, enrollment source, BMI category, physical activity, smoking status, alcohol drinking status, menopausal status, comorbidity index, total energy intake, healthy eating index, and family history of breast cancer. *P*_-trend_ obtained by using the medians of quintiles as a continuous variable in the Cox proportional hazards models.

Abbreviations: CI, confidence Interval; HR, hazard ratio.

### Stratified analyses by hormonal receptor status of BC, menopausal status, race, and BMI

In stratified analyses by ER/PR status (Table 3), the inverse associations between total polyphenols and phenolic acids with risk of BC remained significant and became stronger for the ER+ and PR+ BC type (HR 0.69; 95% CI: 0.51, 0.94; *P*_-trend_ = 0.003 for total polyphenols; HR 0.70; 95% CI: 0.53, 0.95; *P*_-trend_ = 0.005 for phenolic acids) comparing the highest to the lowest quintile. In contrast, the associations were not present among female individuals with ER+ or PR+, ER– and PR–, as well as triple-negative BC (TNBC) (*P*_-heterogeneity_ = 0.11 and 0.35 for total polyphenols and phenolic acids, respectively, among 3 subgroups: ER+ and PR+, ER+ or PR+, ER– and PR–). We conducted a stratified analysis among human epidermal growth factor receptor 2 (HER2) positive and negative participants. There was evidence of a trend toward an inverse association between total polyphenol intakes and BC risk only among HER2-negative female individuals (*P-*_trend_ = 0.04) (Supplemental Table 2).

**TABLE 3:** Associations between polyphenol intakes and breast cancer risk in the Southern Community Cohort Study stratified by hormone receptor status, menopausal status, race, BMI category (*n* = 42,260).

Polyphenol	Quintile 1 (lowest)		Quintile 2		Quintile 3		Quintile 4		Quintile 5 (highest)		*P*_-trend_
	HR	Cases	HR (95% CI)	Cases	HR (95% CI)	Cases	HR (95% CI)	Cases	HR (95% CI)	Cases	
ER+ and PR+											
Total polyphenols	1.00 (ref)	117	1.14 (0.88, 1.47)	136	0.88 (0.67, 1.15)	115	0.97 (0.74, 1.27)	145	0.69 (0.51, 0.94)	99	0.003
Flavonoids	1.00 (ref)	106	1.17 (0.90, 1.54)	128	1.16 (0.89, 1.53)	139	1.02 (0.76, 1.36)	124	0.93 (0.70, 1.25)	115	0.17
Flavones	1.00 (ref)	100	1.17 (0.89, 1.54)	127	1.15 (0.87, 1.52)	130	1.05 (0.78, 1.41)	124	1.02 (0.75, 1.39)	131	0.60
Phenolic acids	1.00 (ref)	126	0.88 (0.68, 1.15)	112	1.11 (0.86, 1.43)	152	0.77 (0.59, 1.01)	112	0.70 (0.53, 0.95)	110	0.005
Lignans	1.00 (ref)	100	1.12 (0.85, 1.47)	127	1.11 (0.83, 1.48)	133	1.05 (0.77, 1.43)	132	0.94 (0.66, 1.34)	120	0.43
Stilbenes	1.00 (ref)	102	1.03 (0.77, 1.39)	118	1.09 (0.80, 1.50)	131	1.11 (0.81, 1.52)	134	1.01 (0.73, 1.41)	127	0.83
Other polyphenols											
Alkylphenols	1.00 (ref)	108	0.91 (0.69, 1.20)	101	1.06 (0.81, 1.40)	131	1.11 (0.84, 1.46)	135	1.06 (0.79, 1.41)	137	0.69
Tyrosols	1.00 (ref)	123	0.85 (0.65, 1.11)	114	0.93 (0.71, 1.21)	119	0.87 (0.66, 1.14)	115	1.09 (0.81, 1.46)	141	0.26
ER+ or PR+											
Total polyphenols	1.00 (ref)	21	1.27 (0.70, 2.29)	28	1.64 (0.93, 2.88)	37	1.20 (0.65, 2.22)	30	1.50 (0.80, 2.83)	32	0.37
Flavonoids	1.00 (ref)	21	1.45 (0.82, 2.56)	34	1.13 (0.62, 2.07)	27	1.53 (0.85, 2.77)	34	1.50 (0.82, 2.74)	32	0.30
Flavones	1.00 (ref)	19	2.00 (1.11, 3.62)	35	1.45 (0.77, 2.74)	26	1.97 (1.06, 3.68)	39	1.38 (0.69, 2.76)	29	0.86
Phenolic acids	1.00 (ref)	24	1.26 (0.72, 2.18)	33	0.83 (0.45, 1.53)	23	1.21 (0.70, 2.08)	35	1.23 (0.67, 2.24)	33	0.50
Lignans	1.00 (ref)	31	0.90 (0.52, 1.54)	29	0.91 (0.52, 1.62)	29	0.83 (0.44, 1.55)	28	1.12 (0.57, 2.21)	31	0.63
Stilbenes	1.00 (ref)	26	0.98 (0.54, 1.79)	28	1.16 (0.62, 2.19)	34	1.06 (0.56, 2.03)	29	1.15 (0.60, 2.21)	31	0.67
Other polyphenols											
Alkylphenols	1.00 (ref)	27	0.97 (0.56, 1.68)	28	1.17 (0.68, 2.01)	33	1.01 (0.57, 1.79)	31	0.98 (0.54, 1.78)	29	0.78
Tyrosols	1.00 (ref)	24	1.36 (0.78, 2.38)	30	1.30 (0.73, 2.32)	27	1.45 (0.82, 2.58)	29	1.91 (1.03, 3.54)	38	0.05
ER- and PR-											
Total polyphenols	1.00 (ref)	57	1.21 (0.83, 1.75)	70	1.04 (0.70, 1.53)	60	0.89 (0.58, 1.36)	48	0.99 (0.63, 1.56)	44	0.58
Flavonoids	1.00 (ref)	56	1.02 (0.70, 1.50)	63	0.85 (0.57, 1.28)	55	0.94 (0.63, 1.42)	61	0.81 (0.52, 1.25)	44	0.31
Flavones	1.00 (ref)	41	1.38 (0.91, 2.10)	59	1.39 (0.91, 2.13)	64	1.36 (0.87, 2.12)	61	1.13 (0.70, 1.82)	54	0.87
Phenolic acids	1.00 (ref)	52	1.29 (0.88, 1.89)	66	1.20 (0.80, 1.79)	62	1.15 (0.77, 1.72)	54	1.20 (0.77, 1.88)	45	0.81
Lignans	1.00 (ref)	47	1.44 (0.97, 2.15)	65	1.41 (0.92, 2.17)	59	1.58 (1.01, 2.49)	65	1.22 (0.72, 2.09)	43	0.74
Stilbenes	1.00 (ref)	61	0.53 (0.34, 0.84)	36	0.88 (0.56, 1.36)	65	0.74 (0.47, 1.18)	56	0.88 (0.56, 1.39)	61	0.64
Other polyphenols											
Alkylphenols	1.00 (ref)	51	0.96 (0.64, 1.45)	46	1.12 (0.74, 1.68)	53	1.61 (1.08, 2.37)	73	1.17 (0.75, 1.80)	56	0.69
Tyrosols	1.00 (ref)	53	1.11 (0.76, 1.63)	62	1.06 (0.71, 1.58)	55	1.08 (0.72, 1.62)	54	1.35 (0.87, 2.09)	55	0.20
TNBC											
Total polyphenols	1.00 (ref)	37	1.16 (0.73, 1.84)	44	1.28 (0.81, 2.04)	46	0.86 (0.50, 1.45)	30	1.01 (0.588, 1.77)	29	0.68
Flavonoids	1.00 (ref)	39	0.89 (0.55, 1.43)	38	0.95 (0.59, 1.53)	41	0.92 (0.56, 1.52)	41	0.72 (0.42, 1.23)	27	0.25
Flavones	1.00 (ref)	25	1.84 (1.09, 3.11)	45	1.58 (0.91, 2.73)	41	1.56 (0.89, 2.75)	39	1.38 (0.75, 2.52)	36	0.96
Phenolic acids	1.00 (ref)	32	1.42 (0.87, 2.30)	42	1.55 (0.95, 2.53)	46	1.25 (0.75, 2.09)	34	1.51 (0.87, 2.62)	32	0.46
Lignans	1.00 (ref)	31	1.63 (1.00, 2.67)	45	1.61 (0.95, 2.71)	42	1.65 (0.94, 2.89)	42	1.17 (0.60, 2.28)	26	0.92
Stilbenes	1.00 (ref)	39	0.76 (0.44, 1.30)	29	1.19 (0.70, 2.03)	48	0.76 (0.42, 1.36)	33	0.97 (0.55, 1.73)	37	0.81
Other polyphenols											
Alkylphenols	1.00 (ref)	30	1.31 (0.79, 2.19)	35	1.36 (0.81, 2.29)	36	1.87 (1.13, 3.10)	48	1.41 (0.81, 2.45)	37	0.68
Tyrosols	1.00 (ref)	34	1.19 (0.74, 1.91)	42	1.31 (0.81, 2.11)	40	1.02 (0.61, 1.71)	31	1.66 (0.97, 2.82)	39	0.10
Premenopausal											
Total polyphenols	1.00 (ref)	82	0.95 (0.68, 1.32)	72	1.03 (0.73, 1.45)	64	0.85 (0.58, 1.26)	48	1.01 (0.67, 1.54)	48	0.99
Flavonoids	1.00 (ref)	80	0.68 (0.47, 0.96)	56	0.84 (0.59, 1.20)	63	0.94 (0.65, 1.35)	66	0.84 (0.57, 1.25)	49	0.99
Flavones	1.00 (ref)	58	1.31 (0.91, 1.89)	68	1.16 (0.79, 1.71)	59	1.56 (1.06, 2.29)	75	1.13 (0.72, 1.76)	54	0.60
Phenolic acids	1.00 (ref)	68	1.27 (0.91, 1.78)	82	1.19 (0.83, 1.71)	68	1.17 (0.80, 1.70)	53	1.02 (0.66, 1.58)	43	0.66
Lignans	1.00 (ref)	72	1.16 (0.82, 1.64)	71	1.25 (0.87, 1.80)	73	1.02 (0.67, 1.54)	55	0.90 (0.55, 1.46)	43	0.42
Stilbenes	1.00 (ref)	76	0.71 (0.48, 1.05)	51	0.79 (0.52, 1.19)	55	0.92 (0.61, 1.39)	58	0.97 (0.65, 1.43)	74	0.55
Other polyphenols											
Alkylphenols	1.00 (ref)	73	0.91 (0.64, 1.29)	60	1.07 (0.76, 1.53)	64	1.20 (0.83, 1.72)	64	1.10 (0.74, 1.64)	53	0.59
Tyrosols	1.00 (ref)	65	0.75 (0.51, 1.10)	47	0.89 (0.61, 1.28)	59	0.80 (0.55, 1.18)	56	1.16 (0.78, 1.72)	87	0.16
Postmenopausal											
Total polyphenols	1.00 (ref)	132	1.22 (0.96, 1.54)	176	0.97 (0.76, 1.24)	163	1.03 (0.81, 1.31)	197	0.84 (0.64, 1.09)	154	0.02
Flavonoids	1.00 (ref)	117	1.48 (1.16, 1.88)	187	1.16 (0.89, 1.49)	168	1.18 (0.92, 1.53)	180	1.13 (0.87, 1.46)	170	0.48
Flavones	1.00 (ref)	114	1.35 (1.05, 1.73)	174	1.23 (0.96, 1.59)	173	1.10 (0.84, 1.43)	169	1.15 (0.87, 1.51)	192	0.76
Phenolic acids	1.00 (ref)	153	0.86 (0.67, 1.09)	143	1.03 (0.82, 1.29)	191	0.87 (0.69, 1.09)	175	0.76 (0.59, 0.97)	160	0.02
Lignans	1.00 (ref)	119	1.15 (0.90, 1.48)	164	1.09 (0.84, 1.42)	163	1.25 (0.95, 1.64)	200	1.11 (0.81, 1.51)	176	0.69
Stilbenes	1.00 (ref)	137	0.90 (0.70, 1.18)	149	1.09 (0.83, 1.43)	197	0.95 (0.71, 1.25)	175	0.96 (0.72, 1.28)	164	0.79
Other polyphenols											
Alkylphenols	1.00 (ref)	129	0.94 (0.73, 1.21)	129	1.07 (0.83, 1.36)	168	1.24 (0.98, 1.59)	204	1.07 (0.83, 1.38)	192	0.80
Tyrosols	1.00 (ref)	155	1.04 (0.83, 1.31)	174	1.03 (0.81, 1.29)	158	1.08 (0.85, 1.36)	162	1.26 (0.98, 1.63)	173	0.05
NHB											
Total polyphenols	1.00 (ref)	185	1.07 (0.86, 1.31)	209	0.90 (0.72, 1.12)	181	0.93 (0.73, 1.18)	155	0.81 (0.59, 1.10)	59	0.09
Flavonoids	1.00 (ref)	154	1.19 (0.95, 1.48)	194	0.97 (0.77, 1.23)	167	1.06 (0.83, 1.35)	174	1.01 (0.76, 1.33)	100	0.65
Flavones	1.00 (ref)	126	1.30 (1.02, 1.66)	171	1.17 (0.91, 1.50)	158	1.08 (0.83, 1.40)	154	1.07 (0.82, 1.41)	180	0.55
Phenolic acids	1.00 (ref)	185	0.88 (0.71, 1.10)	168	1.08 (0.87, 1.34)	208	0.93 (0.74, 1.16)	173	0.73 (0.53, 1.00)	55	0.06
Lignans	1.00 (ref)	185	1.16 (0.94, 1.43)	214	1.03 (0.81, 1.31)	170	1.10 (0.85, 1.42)	147	0.95 (0.69, 1.31)	73	0.61
Stilbenes	1.00 (ref)	172	0.78 (0.61, 1.00)	142	0.91 (0.70, 1.18)	167	0.85 (0.65, 1.12)	149	1.01 (0.77, 1.31)	159	0.32
Other polyphenols											
Alkylphenols	1.00 (ref)	181	0.88 (0.71, 1.11)	141	1.00 (0.79, 1.25)	151	1.23 (0.98, 1.54)	173	0.95 (0.74, 1.21)	143	0.75
Tyrosols	1.00 (ref)	185	0.97 (0.78, 1.21)	174	0.92 (0.73, 1.16)	137	1.01 (0.80, 1.27)	142	1.34 (1.03, 1.73)	151	0.01
NHW											
Total polyphenols	1.00 (ref)	21	1.83 (1.04, 3.21)	35	1.43 (0.81, 2.52)	33	1.54 (0.93, 2.55)	87	1.35 (0.83, 2.19)	136	0.71
Flavonoids	1.00 (ref)	37	1.11 (0.71, 1.75)	44	1.26 (0.81, 1.97)	56	1.13 (0.72, 1.75)	60	1.11 (0.75, 1.64)	115	0.98
Flavones	1.00 (ref)	43	1.46 (0.98, 2.16)	68	1.38 (0.92, 2.07)	68	1.56 (1.03, 2.36)	80	1.28 (0.79, 2.05)	53	0.54
Phenolic acids	1.00 (ref)	34	1.26 (0.79, 2.02)	42	1.01 (0.63, 1.63)	43	1.12 (0.71, 1.76)	54	0.99 (0.66, 1.47)	139	0.42
Lignans	1.00 (ref)	5	1.56 (0.52, 4.66)	17	2.59 (0.93, 7.20)	57	2.63 (0.95, 7.29)	99	2.40 (0.85, 6.74)	134	0.40
Stilbenes	1.00 (ref)	35	1.08 (0.67, 1.75)	54	1.33 (0.81, 2.20)	80	1.12 (0.67, 1.87)	76	0.80 (0.47, 1.38)	67	0.06
Other polyphenols											
Alkylphenols	1.00 (ref)	16	1.39 (0.77, 2.53)	41	1.74 (0.99, 3.07)	74	1.89 (1.07, 3.33)	92	1.92 (1.07, 3.44)	89	0.21
Tyrosols	1.00 (ref)	32	0.78 (0.48, 1.25)	40	1.04 (0.68, 1.60)	75	0.85 (0.55, 1.32)	66	1.03 (0.66, 1.59)	99	0.51
BMI <25 kg/m^2^											
Total polyphenols	1.00 (ref)	20	1.45 (0.83, 2.55)	36	1.30 (0.73, 2.33)	30	1.32 (0.74, 2.37)	39	1.31 (0.72, 2.37)	50	0.77
Flavonoids	1.00 (ref)	31	0.97 (0.59, 1.59)	37	0.66 (0.38, 1.14)	25	0.93 (0.56, 1.56)	39	0.95 (0.56, 1.59)	43	0.83
Flavones	1.00 (ref)	24	1.72 (1.02, 2.90)	40	1.48 (0.85, 2.58)	34	1.62 (0.92, 2.83)	40	1.55 (0.85, 2.81)	37	0.44
Phenolic acids	1.00 (ref)	19	1.41 (0.79, 2.49)	35	1.34 (0.75, 2.39)	37	1.31 (0.73, 2.35)	31	1.26 (0.71, 2.26)	53	0.96
Lignans	1.00 (ref)	18	1.44 (0.77, 2.70)	25	1.93 (1.05, 3.55)	37	1.98 (1.05, 3.75)	43	1.96 (0.98, 3.89)	52	0.14
Stilbenes	1.00 (ref)	31	0.96 (0.53, 1.74)	23	1.68 (0.95, 2.96)	40	0.94 (0.51, 1.72)	28	1.25 (0.71, 2.19)	53	0.56
Other polyphenols											
Alkylphenols	1.00 (ref)	24	1.17 (0.67, 2.02)	30	1.21 (0.70, 2.11)	33	1.76 (1.04, 3.01)	48	1.40 (0.78, 2.51)	40	0.57
Tyrosols	1.00 (ref)	25	1.14 (0.64, 2.00)	26	1.24 (0.72, 2.15)	32	1.16 (0.67, 2.00)	37	1.08 (0.60, 1.95)	55	0.93
BMI ≥25 kg/m^2^											
Total polyphenols	1.00 (ref)	190	1.06 (0.86, 1.30)	207	0.95 (0.77, 1.17)	198	0.92 (0.74, 1.15)	204	0.79 (0.62, 1.01)	150	0.02
Flavonoids	1.00 (ref)	164	1.17 (0.95, 1.46)	203	1.09 (0.87, 1.36)	203	1.10 (0.88, 1.38)	206	0.99 (0.78, 1.26)	173	0.41
Flavones	1.00 (ref)	147	1.23 (0.99, 1.54)	196	1.18 (0.94, 1.49)	200	1.15 (0.90, 1.45)	202	1.07 (0.83, 1.37)	204	0.72
Phenolic acids	1.00 (ref)	197	0.94 (0.76, 1.17)	187	1.07 (0.87, 1.32)	220	0.91 (0.74, 1.13)	197	0.77 (0.60, 0.98)	148	0.01
Lignans	1.00 (ref)	171	1.08 (0.87, 1.34)	205	1.04 (0.83, 1.30)	198	1.07 (0.84, 1.36)	211	0.92 (0.70, 1.22)	164	0.41
Stilbenes	1.00 (ref)	180	0.81 (0.64, 1.02)	173	0.90 (0.70, 1.16)	210	0.91 (0.71, 1.18)	204	0.89 (0.69, 1.16)	182	0.81
Other polyphenols											
Alkylphenols	1.00 (ref)	173	0.90 (0.72, 1.12)	157	1.06 (0.85, 1.32)	197	1.17 (0.94, 1.45)	219	1.04 (0.82, 1.31)	203	0.75
Tyrosols	1.00 (ref)	191	0.94 (0.76, 1.15)	193	0.96 (0.78, 1.19)	183	0.98 (0.79, 1.21)	177	1.31 (1.04, 1.65)	205	0.004

Abbreviations: CI, confidence interval; ER, estrogen receptor; HR, hazard ratio; NHB, non-Hispanic Black; NHW, non-Hispanic white; PR, progesterone receptor; SCCS, Southern Community Cohort Study; TNBC, triple-negative breast cancer.

Models adjusted for age, race, household income, enrollment source, BMI category, physical activity, smoking status, alcohol drinking status, menopausal status, comorbidity index, total energy intake, healthy eating index, and family history of breast cancer unless a particular variable was being stratified. *P*_-trend_ obtained by using the medians of quintiles as a continuous variable in the Cox proportional hazards models.

In stratified analyses by menopausal status, there was evidence of a trend toward an inverse association between total polyphenols and risk of BC among postmenopausal female individuals (HR 0.84; 95% CI: 0.64, 1.09; *P*_-trend_ = 0.02). The significant inverse association between phenolic acid intake and BC risk was only present among postmenopausal female individuals (HR 0.76; 95% CI: 0.59, 0.97; *P*_-trend_ = 0.02), but not premenopausal female individuals (*β* = –0.03, *P*_-interaction_ = 0.55).

Stratified analyses by racial and ethnic subgroups showed that the inverse association between intakes of phenolic acids and risk of BC was of borderline significance among NHB female individuals (HR 0.73; 95% CI: 0.53, 1.00; *P*_-trend_ = 0.06) whereas tyrosols were significantly associated with increased risk of BC (HR 1.34; 95% CI: 1.03, 1.73; *P*_-trend_ = 0.01) (*β* = –0.07, *P*_-interaction_ = 0.09 for phenolic acids; *β* = 0.05, *P*_-interaction_ = 0.22 for tyrosols). In contrast, no associations were observed for intakes of total polyphenols, classes, and subclasses of polyphenols among NHW female individuals.

In stratified analyses by BMI categories (<25 kg/m^2^ compared with ≥25 kg/m^2^), there was evidence of a trend toward an inverse association between intakes of total polyphenols and BC risk among female individuals with BMI ≥25 kg/m^2^ (HR 0.79; 95% CI: 0.62, 1.01; *P*_-trend_ = 0.02). The inverse association between intakes of phenolic acids and risk of BC remained significant and became more pronounced only among female individuals with BMI ≥ 25 kg/m^2^ (HR 0.77; 95% CI: 0.60, 0.98; *P*_-trend_ = 0.01) (*β* = –0.03, *P*_-interaction_ = 0.39). Similar to the main analysis, intakes of tyrosols were associated with increased risk of BC among participants with BMI ≥ 25 kg/m^2^ with the corresponding HR (95% CI) of 1.31 (1.04, 1.65) (*P*_-trend_ = 0.004) (*β* = –0.01, *P*_-interaction_ = 0.71). In contrast, intakes of total polyphenols, classes, and subclasses of polyphenols were not associated with the risk of BC among participants with BMI < 25 kg/m^2^.

### Stratified analyses by intakes of phenolic acids and tyrosols

Because we observed a significant inverse association for phenolic acids and a positive association for tyrosols, further stratified analyses were conducted to examine whether phenolic aids and tyrosols interacted with each other in relation to the risk of BC (Table 4). Although the interaction was not significant, in the subsequent stratified analyses in low and high levels by the median intakes, there was evidence of a trend toward an inverse association between phenolic acids and BC risk in those with the intake of tyrosols ≥ median (HR 0.81; 95% CI: 0.59, 1.10; *P*_-trend_ = 0.07), but not in those with the intake of tyrosols less than the median. In contrast, the trend toward a positive association between tyrosols and risk of BC was only present in those with the intake of phenolic acids less than the median (HR 1.33; 95% CI: 0.99, 1.79; *P*_-trend_ = 0.02), but not in those with intakes of phenolic acids ≥ median.

**TABLE 4:** Stratified analyses in associations between intakes of phenolic acids, tyrosols, and breast cancer risk in the Southern Community Cohort Study (*n* = 42,260).

Polyphenol	Quintile 1 (lowest)		Quintile 2		Quintile 3		Quintile 4		Quintile 5 (highest)		*P* linear trend^2^	*P* for interaction
	HR	Cases	HR (95% CI)	Cases	HR (95% CI)	Cases	HR (95% CI)	Cases	HR (95% CI)	Cases		
Phenolic acids												
Tyrosols low	1.00 (ref)	131	0.86 (0.66, 1.14)	96	1.15 (0.88, 1.49)	132	0.80 (0.61, 1.05)	107	0.86 (0.64, 1.17)	87	0.23	0.53 (*β* = –0.03)
Tyrosols high	1.00 (ref)	91	1.08 (0.81, 1.43)	129	1.00 (0.75, 1.34)	128	1.10 (0.83, 1.47)	124	0.81 (0.59, 1.10)	116	0.07	
Tyrosols												
Phenolic acids low	1.00 (ref)	119	0.90 (0.69, 1.18)	120	0.98 (0.75, 1.28)	117	0.95 (0.72, 1.25)	103	1.33 (0.99, 1.79)	124	0.02	0.75 (*β* = –0.01)
Phenolic acids high	1.00 (ref)	102	1.02 (0.76, 1.35)	102	1.00 (0.75, 1.34)	101	1.04 (0.78, 1.38)	116	1.18 (0.86, 1.61)	137	0.24	

Abbreviations: CI, confidence interval; HR, hazard ratio.

Low and high levels of phenolic acids and tyrosols were classified by medium levels. Models adjusted for age, race, household income, enrollment source, BMI category, physical activity, smoking status, alcohol drinking status, menopausal status, comorbidity index, total energy intake, healthy eating index, and family history of breast cancer.

### Sensitivity analyses

We observed similar patterns in the sensitivity analyses by excluding those who were followed up <12 mo (Supplemental Table 3).

## Discussion

In one of the largest prospective cohort studies with predominantly low-income participants designed to evaluate racial and socioeconomic cancer disparities, we found that intake of total polyphenols was much higher in NHW female individuals than in NHB female individuals. Also, we demonstrated that intakes of total polyphenols, particularly phenolic acids, were associated with reduced risk of BC incidence among female individuals with the ER+ and PR+ BC types. Phenolic acid was inversely related to BC among postmenopausal female individuals and female individuals with a BMI ≥ 25 kg/m^2^. On the other hand, intakes of tyrosols were associated with an increased risk of BC among NHB female individuals and female individuals with a BMI ≥ 25 kg/m^2^.

Polyphenols have a wide range of biological activities. Some polyphenols possess antioxidant [36,37], anti-inflammatory [38,39], and cancer inhibitory [40,41] properties, mainly through induction of apoptosis, cell cycle arrest, and inhibition of angiogenesis via modulation of various signal transduction pathways and transcription factors [40]. However, as estrogen plays a key role in the development of BC through ER, particularly in postmenopausal female individuals [3], the estrogenic or antiestrogenic effects are more relevant to the etiology. With a phenolic ring, phenolic aids were shown to inhibit aromatases [13,14]. Aromatases convert androgens to estrogens in fat, particularly postmenopausal female individuals [42,43]. Thus, the potential mechanism is that phenolic acids may inhibit aromatases in fat and, in turn, decrease levels of estrogens in postmenopausal female individuals. As such, our findings are biologically plausible and internally consistent that the inverse associations with higher intakes of total polyphenols, particularly phenolic acids primarily occurred among female individuals with higher BMI, among postmenopausal female individuals, or for the ER+ and PR+ BC type. This finding on total polyphenols is consistent with that reported from the SUN cohort conducted in Spain [18]. Also, the SUN cohort is the only previous cohort study linking intakes of phenolic acids with a reduced risk of BC among postmenopausal female individuals (third tertile compared with first tertile HR 0.37, 95% CI: 0.16, 0.85; *P*_-trend_ = 0.029 for hydroxycinnamic acids intake; HR 0.33, 95% CI: 0.14, 0.78; *P*_-trend_ = 0.012 for chlorogenic acids intake) [17]. According to our previous analysis, phenolic acids accounted for the largest, or 51% of total polyphenols whereas regular coffee intake accounted for the largest, or 59% of phenolic acids intake [8]. Although not entirely consistent [22,[44], [45], [46], [47]], pooled analyses, systematic reviews, and meta-analysis consistently found that coffee is inversely associated with BC risk in postmenopausal female individuals [[48], [49], [50], [51], [52]]. Therefore, these findings add further support to the potential beneficial effects of phenolic acids for the prevention of BC. The findings from the current study also showed that the inverse association seemed to primarily exist in NHB female individuals whereas average intakes of polyphenols in NHW female individuals were double that in NHB female individuals.

In contrast to phenolic acids, previous studies showed that tyrosol-related molecules may upregulate estrogen-related receptor α [53]. Although this effect could be beneficial to bone health [54] and the prevention of colorectal cancer [8], this effect may provide a possible explanation that higher intakes of tyrosols, mainly derived from olives and olive oil [8,28], were associated with an increased risk of BC, particularly among NHB female individuals and female individuals with BMI ≥ 25 kg/m^2^. Tyrosols activate estrogen-related receptor α which plays a key role in the development of BC. Estrogen-related receptor α is involved in upregulating pathways like glycolysis and mitochondrial respiration which supports tumor growth and progression [55,56]. When the intake levels of phenolic acids were high, the increased risk of BC associated with intakes of tyrosols disappeared. Although our findings should be confirmed in future studies, they indicate that consumption of phenolic acids could minimize the potential adverse effects of tyrosols on BC risk.

Unlike phenolic acids, previous studies have focused on flavonoids, 1 major class of polyphenols, but suggest no association between flavonoids and the risk of BC. Soy isoflavones, with a similar structure to estrogens [11], are a subclass of polyphenols belonging to flavonoids. Previous research demonstrated that soy isoflavones inhibited 17beta-hydroxysteroid dehydrogenase type I [57,58], a key enzyme in catalyzing estrone (E1), to the biologically more active estradiol (E2) [59]. However, prospective studies showed the inverse associations between intakes of soy isoflavones and risk of BC were observed among studies in the East Asian population, a population consuming the highest levels of soy foods, but not in studies conducted in Western societies [60]. In East Asian female individuals, the inverse association between isoflavones and risk of BC was primarily present in female individuals with a higher BMI or for ER/PR-positive BC [10,19,20]. Collectively, it is possible that high doses of isoflavones are required to reduce the risk of BC, particularly among female individuals with overweight/obesity and for ER/PR-positive BC. Another group of polyphenols with a similar structure to estrogens within the class of flavonoids is lignan. However, most previous prospective studies did not find an association [16,[61], [62], [63]] whereas 2 studies found an inverse association [64] and the association was primarily for ER/PR-positive BC among postmenopausal female individuals [64].

With a catechol ring, flavanols and flavonols are the only 2 common groups of polyphenols possessing a similar structure to catechol estrogen metabolites [11]. Flavanols and flavonols are 2 major subclasses of polyphenols belonging to the class of flavonoids. Two cohort studies have examined the associations between bioavailable flavonoids and the risk of BC. In the first report, urinary excretions of flavanols and flavonols were not related to the risk of BC in a prospective cohort study [11] which was supported by a subsequent cohort study measuring plasma levels [15]. Likewise, 7 of 8 prospective cohort studies failed to find an association between intake of total flavonoids and risk of BC [16,[65], [66], [67], [68], [69], [70]] except for 1 [71].

The strengths of this study include the large sample size, the prospective cohort design, and the ability to represent a large geographic area in the United States. Furthermore, this study was conducted in one of the largest prospective cohort studies designed to study the root causes of cancer disparities for low-income and NHB populations in the United States. A major advantage of this study over many past studies of polyphenols was the specific matching of nearly 2000 foods to potential polyphenol entries that also captured both sex and racial intake differences; additionally, we used aglycone equivalents in the analysis which may be more representative of polyphenol content after digestion. Finally, the linkage of the cohort to the National Death Index and state cancer registries allowed for nearly complete follow-up of participants, and those who were ultimately lost to follow-up were accounted for by censoring at the age of the last follow-up.

There are also several limitations to this study. First, although the FFQ has been validated, FFQs in general may be subject to misclassification in measuring dietary intakes and can be prone to bias in the form of inaccurate recall or intentional misreporting. However, recall bias is minimized as this is a longitudinal cohort in which diet assessment occurred before cancer diagnosis. A second limitation is that diet and other potential confounders were only assessed at baseline; a single measure of dietary intake may not reflect their long-term usual diet over the follow-up period. We also attempted to account for reverse causation by conducting sensitivity analysis excluding participants who were followed up <12 mo and found similar results. A third limitation of the study is the potential for residual confounding. We attempted to account for this by adjusting for variables that were associated with both exposure and outcome; however, it is possible that the variables included in our model did not completely account for confounding due to some unknown other confounders. A fourth limitation of this study is generalizability; this cohort represents a low socioeconomic status population of predominantly NHB individuals in the southeastern United States. Therefore, the results of this study may be culturally, geographically, or socioeconomically bound and may not generalize to populations outside the United States.

In conclusion, in one of the largest prospective cohort studies designed to evaluate racial and socioeconomic cancer disparities, intakes of total polyphenols, particularly phenolic acids, were associated with reduced risk of BC incidence among female individuals with the ER+ and PR+ BC type. Phenolic acid was inversely related to BC among postmenopausal female individuals and female individuals with a BMI≥25 kg/m^2^. On the other hand, intakes of tyrosols were associated with an increased risk of BC among NHB female individuals and female individuals with a BMI ≥25 kg/m^2^. Future studies are warranted to confirm our findings.

## Author contributions

The authors’ responsibilities were as follows – MJS, QD: designed research and primary responsibility for final content; LTF, LF, HM, MJS, QD: conducted research; LF, LTF: analyzed data; LF, LTF, HM, DY, MJS, QD: drafted this paper; and all authors: read and approved the final manuscript.

## Disclaimer

The content is solely the responsibility of the authors and does not necessarily represent the official views of the National Institutes of Health. This manuscript was reviewed by SCCS publications committee for scientific content.

## Data availability

Data described in the manuscript, code book, and analytic code will be made available on request after the investigator has obtained approval from the ethnic committee and data-sharing committee of the SCCS.

## Funding

Research reported in this publication was supported by the USDA Award No. 2022-38821-37352 and the National Cancer Institute of the National Institutes of Health under Award Number U01CA202979 and R01CA092447. The content is solely the responsibility of the authors and does not necessarily represent the official views of the National Institutes of Health.

## Conflict of interest

The authors report no conflicts of interest.

## References

[1] Giaquinto A.N., Sung H., Miller K.D., Kramer J.L., Newman L.A., Minihan A. (2022). Breast cancer statistics, 2022. CA Cancer J. Clin..

[2] Giaquinto A.N., Miller K.D., Tossas K.Y., Winn R.A., Jemal A., Siegel R.L. (2022). Cancer statistics for African American/Black people 2022. CA Cancer J. Clin..

[3] Yager J.D., Davidson N.E. (2006). Estrogen carcinogenesis in breast cancer. N. Engl. J. Med..

[4] Rauscher G.H., Silva A., Pauls H., Frasor J., Bonini M.G., Hoskins K. (2017). Racial disparity in survival from estrogen and progesterone receptor-positive breast cancer: implications for reducing breast cancer mortality disparities. Breast Cancer Res. Treat..

[5] Ma H., Lu Y., Malone K.E., Marchbanks P.A., Deapen D.M., Spirtas R. (2013). Mortality risk of black women and white women with invasive breast cancer by hormone receptors, HER2, and p53 status. BMC Cancer.

[6] Iqbal J., Ginsburg O., Rochon P.A., Sun P., Narod S.A. (2015). Differences in breast cancer stage at diagnosis and cancer-specific survival by race and ethnicity in the United States. JAMA.

[7] González-Vallinas M., González-Castejón M., Rodríguez-Casado A., Ramírez de Molina A. (2013). Dietary phytochemicals in cancer prevention and therapy: a complementary approach with promising perspectives. Nutr. Rev..

[8] Fike L.T., Munro H., Yu D., Dai Q., Shrubsole M.J. (2022). Dietary polyphenols and the risk of colorectal cancer in the prospective Southern Community Cohort Study. Am. J. Clin. Nutr..

[9] Dai Q., Shu X.O., Jin F., Potter J.D., Kushi L.H., Teas J. (2001). Population-based case-control study of soyfood intake and breast cancer risk in Shanghai. Br. J. Cancer..

[10] Dai Q., Franke A.A., Jin F., Shu X.-O., Hebert J.R., Custer L.J. (2002). Urinary excretion of phytoestrogens and risk of breast cancer among Chinese women in Shanghai. Cancer Epidemiol. Biomark. Prev..

[11] Luo J., Gao Y.-T., Chow W.-H., Shu X.-O., Li H., Yang G. (2010). Urinary polyphenols and breast cancer risk: results from the Shanghai Women’s Health Study. Breast Cancer Res. Treat..

[12] Cipolletti M., Solar Fernandez V., Montalesi E., Marino M., Fiocchetti M. (2018). Beyond the antioxidant activity of dietary polyphenols in cancer: the modulation of Estrogen Receptors (ERs) signaling. Int. J. Mol. Sci..

[13] Lephart E.D. (2015). Modulation of aromatase by phytoestrogens. Enzyme Res..

[14] Monteiro R., Azevedo I., Calhau C. (2006). Modulation of aromatase activity by diet polyphenolic compounds. J. Agric. Food Chem..

[15] Iwasaki M., Inoue M., Sasazuki S., Miura T., Sawada N., Yamaji T. (2010). Plasma tea polyphenol levels and subsequent risk of breast cancer among Japanese women: a nested case-control study. Breast Cancer Res. Treat..

[16] Zamora-Ros R., Ferrari P., González C.A., Tjønneland A., Olsen A., Bredsdorff L. (2013). Dietary flavonoid and lignan intake and breast cancer risk according to menopause and hormone receptor status in the European Prospective Investigation into Cancer and Nutrition (EPIC) Study. Breast Cancer Res. Treat..

[17] Romanos-Nanclares A., Sánchez-Quesada C., Gardeazábal I., Martínez-González M.Á., Gea A., Toledo E. (2020). Phenolic acid subclasses, individual compounds, and breast cancer risk in a Mediterranean cohort: the SUN project. J. Acad. Nutr. Diet..

[18] Gardeazabal I., Romanos-Nanclares A., Martínez-González M.Á., Sánchez-Bayona R., Vitelli-Storelli F., Gaforio J.J. (2019). Total polyphenol intake and breast cancer risk in the Seguimiento Universidad de Navarra (SUN) cohort. Br. J. Nutr..

[19] Zheng W., Dai Q., Custer L.J., Shu X.O., Wen W.Q., Jin F. (1999). Urinary excretion of isoflavonoids and the risk of breast cancer. Cancer Epidemiol. Biomarkers Prev..

[20] Chen Z., Zheng W., Custer L.J., Dai Q., Shu X.O., Jin F. (1999). Usual dietary consumption of soy foods and its correlation with the excretion rate of isoflavonoids in overnight urine samples among Chinese women in Shanghai. Nutr. Cancer..

[21] Boggs D.A., Palmer J.R., Wise L.A., Spiegelman D., Stampfer M.J., Adams-Campbell L.L. (2010). Fruit and vegetable intake in relation to risk of breast cancer in the black women’s health study. Am. J. Epidemiol..

[22] Boggs D.A., Palmer J.R., Stampfer M.J., Spiegelman D., Adams-Campbell L.L., Rosenberg L. (2010). Tea and coffee intake in relation to risk of breast cancer in the Black Women’s Health Study. Cancer Causes Control.

[23] Signorello L.B., Hargreaves M.K., Steinwandel M.D., Zheng W., Cai Q., Schlundt D.G. (2005). Southern community cohort study: establishing a cohort to investigate health disparities. J. Natl. Med. Assoc..

[24] Buchowski M.S., Schlundt D.G., Hargreaves M.K., Hankin J.H., Signorello L.B., Blot W.J. (2003). Development of a culturally sensitive food frequency questionnaire for use in the Southern Community Cohort Study. Cell Mol. Biol. (Noisy-le-grand)..

[25] Signorello L.B., Munro H.M., Buchowski M.S., Schlundt D.G., Cohen S.S., Hargreaves M.K. (2009). Estimating nutrient intake from a food frequency questionnaire: incorporating the elements of race and geographic region. Am. J. Epidemiol..

[26] Bhagwat S., Haytowitz D.B., Wasswa-Kintu S. (2015). USDA’s expanded flavonoid database for the assessment of dietary intakes, release 1.1 - December 2015[Internet]. Nutrient Data Laboratory, Beltsville Human Nutrition Research Center, ARS, USDA.

[27] Available N. (2015). USDA database for the proanthocyanidin content of selected foods, release 2 (2015) [Internet]. Nutrient Data Laboratory, Beltsville Human Nutrition Research Center, ARS, USDA.

[28] Neveu V., Perez-Jiménez J., Vos F., Crespy V., du Chaffaut L., Mennen L. (2010). Phenol-explorer: an online comprehensive database on polyphenol contents in foods. Database (Oxford).

[29] Pérez-Jiménez J., Neveu V., Vos F., Scalbert A. (2010). Systematic analysis of the content of 502 polyphenols in 452 foods and beverages: an application of the phenol-explorer database. J. Agric. Food Chem..

[30] Willett W.C., Howe G.R., Kushi L.H. (1997). Adjustment for total energy intake in epidemiologic studies. Am. J. Clin. Nutr..

[31] Charlson M.E., Pompei P., Ales K.L., MacKenzie C.R. (1987). A new method of classifying prognostic comorbidity in longitudinal studies: development and validation. J. Chronic Dis..

[32] Sonderman J.S., Munro H.M., Blot W.J., Tarone R.E., McLaughlin J.K. (2014). Suicides, homicides, accidents, and other external causes of death among blacks and whites in the Southern Community Cohort Study. PLOS ONE.

[33] Bowman S.A., Friday J.E., Moshfegh A.J. (2008). MyPyramid Equivalents Database, 2.0 for USDA survey foods, 2003–2004: documentation and user guide, US Dep. Agric.

[34] Guenther P.M., Casavale K.O., Reedy J., Kirkpatrick S.I., Hiza H.A.B., Kuczynski K.J. (2013). Update of the healthy eating index: HEI-2010. J. Acad. Nutr. Diet..

[35] Durrleman S., Simon R. (1989). Flexible regression models with cubic splines. Stat. Med..

[36] Bucciantini M., Leri M., Nardiello P., Casamenti F., Stefani M. (2021). Olive polyphenols: antioxidant and anti-inflammatory properties. Antioxidants (Basel).

[37] Magrone T., Magrone M., Russo M.A., Jirillo E. (2019). Recent advances on the anti-inflammatory and antioxidant properties of red grape polyphenols: in vitro and in vivo studies. Antioxidants (Basel).

[38] Pap N., Fidelis M., Azevedo L., do Carmo M.A.V., Wang D., Mocan A. (2021). Berry polyphenols and human health: evidence of antioxidant, anti-inflammatory, microbiota modulation, and cell-protecting effects. Curr. Opin. Food Sci..

[39] Nani A., Murtaza B., Sayed Khan A., Khan N.A., Hichami A. (2021). Antioxidant and anti-inflammatory potential of polyphenols contained in Mediterranean diet in obesity: molecular mechanisms. Molecules.

[40] Abbaszadeh H., Keikhaei B., Mottaghi S. (2019). A review of molecular mechanisms involved in anticancer and antiangiogenic effects of natural polyphenolic compounds. Phytother. Res..

[41] Bhosale P.B., Ha S.E., Vetrivel P., Kim H.H., Kim S.M., Kim G.S. (2020). Functions of polyphenols and its anticancer properties in biomedical research: a narrative review, Transl. Cancer Res..

[42] Dai Q., Franke A.A., Yu H., Shu X.-O., Jin F., Hebert J.R. (2003). Urinary phytoestrogen excretion and breast cancer risk: evaluating potential effect modifiers endogenous estrogens and anthropometrics. Cancer Epidemiol. Biomarkers Prev..

[43] Ishikawa T., Glidewell-Kenney C., Jameson J.L. (2006). Aromatase-independent testosterone conversion into estrogenic steroids is inhibited by a 5 alpha-reductase inhibitor. J. Steroid. Biochem. Mol. Biol..

[44] Zheng K.H., Zhu K., Wactawski-Wende J., Freudenheim J.L., LaMonte M.J., Hovey K.M. (2021). Caffeine intake from coffee and tea and invasive breast cancer incidence among postmenopausal women in the Women’s Health Initiative. Int. J. Cancer..

[45] Yaghjyan L., McLaughlin E., Lehman A., Neuhouser M.L., Rohan T., Lane D.S. (2022). Associations of coffee/caffeine consumption with postmenopausal breast cancer risk and their interactions with postmenopausal hormone use. Eur. J. Nutr..

[46] Bhoo Pathy N., Peeters P., van Gils C., Beulens J.W.J., van der Graaf Y., Bueno-de-Mesquita B. (2010). Coffee and tea intake and risk of breast cancer. Breast Cancer Res. Treat..

[47] Gierach G.L., Freedman N.D., Andaya A., Hollenbeck A.R., Park Y., Schatzkin A. (2012). Coffee intake and breast cancer risk in the NIH-AARP diet and health study cohort. Int. J. Cancer..

[48] Ganmaa D., Willett W.C., Li T.Y., Feskanich D., van Dam R.M., Lopez-Garcia E. (2008). Coffee, tea, caffeine and risk of breast cancer: a 22-year follow-up. Int. J. Cancer..

[49] Nehlig A., Reix N., Arbogast P., Mathelin C. (2021). Coffee consumption and breast cancer risk: a narrative review in the general population and in different subtypes of breast cancer. Eur. J. Nutr..

[50] Jiang W., Wu Y., Jiang X. (2013). Coffee and caffeine intake and breast cancer risk: an updated dose–response meta-analysis of 37 published studies. Gynecol. Oncol..

[51] Wang S., Li X., Yang Y., Xie J., Liu M., Zhang Y. (2021). Does coffee, tea and caffeine consumption reduce the risk of incident breast cancer? A systematic review and network meta-analysis. Public Health Nutr.

[52] Li Y., Ma L. (2021). The association between coffee intake and breast cancer risk: a meta-analysis and dose-response analysis using recent evidence. Ann. Palliat. Med..

[53] Martínez-Lara E., Peña A., Calahorra J., Cañuelo A., Siles E. (2016). Hydroxytyrosol decreases the oxidative and nitrosative stress levels and promotes angiogenesis through HIF-1 independent mechanisms in renal hypoxic cells. Food Funct.

[54] Karković Marković A., Torić J., Barbarić M., Jakobušić Brala C. (2019). Hydroxytyrosol, tyrosol and derivatives and their potential effects on human health. Mol. Basel. Switz..

[55] Cai Q., Lin T., Kamarajugadda S., Lu J. (2013). Regulation of glycolysis and the Warburg effect by estrogen-related receptors. Oncogene.

[56] Chaltel-Lima L., Domínguez F., Domínguez-Ramírez L., Cortes-Hernandez P. (2023). The role of the estrogen-related receptor alpha (ERRa) in hypoxia and its implications for cancer metabolism. Int. J. Mol. Sci..

[57] Adlercreutz H., Mazur W. (1997). Phyto-oestrogens and Western diseases. Ann. Med..

[58] Makela S., Davis V.L., Tally W.C., Korkman J., Salo L., Vihko R. (1994). Dietary estrogens act through estrogen receptor-mediated processes and show no antiestrogenicity in cultured breast cancer cells. Environ. Health Perspect..

[59] Dai Q., Xu W.-H., Long J.-R., Courtney R., Xiang Y.-B., Cai Q. (2007). Interaction of soy and 17beta-HSD1 gene polymorphisms in the risk of endometrial cancer, Pharmacogenet. Genomics.

[60] Dong J.-Y., Qin L.-Q. (2011). Soy isoflavones consumption and risk of breast cancer incidence or recurrence: a meta-analysis of prospective studies. Breast Cancer Res. Treat..

[61] Buck K., Zaineddin A.K., Vrieling A., Linseisen J., Chang-Claude J. (2010). Meta-analyses of lignans and enterolignans in relation to breast cancer risk. Am. J. Clin. Nutr..

[62] Velentzis L.S., Cantwell M.M., Cardwell C., Keshtgar M.R., Leathem A.J., Woodside J.V. (2009). Lignans and breast cancer risk in pre- and post-menopausal women: meta-analyses of observational studies. Br. J. Cancer..

[63] Ward H.A., Kuhnle G.G.C., Mulligan A.A., Lentjes M.A.H., Luben R.N., Khaw K.-T. (2010). Breast, colorectal, and prostate cancer risk in the European Prospective Investigation into Cancer and Nutrition-Norfolk in relation to phytoestrogen intake derived from an improved database. Am. J. Clin. Nutr..

[64] Touillaud M.S., Thiébaut A.C.M., Fournier A., Niravong M., Boutron-Ruault M.-C., Clavel-Chapelon F. (2007). Dietary lignan intake and postmenopausal breast cancer risk by estrogen and progesterone receptor status. J. Natl. Cancer Inst..

[65] Knekt P., Kumpulainen J., Järvinen R., Rissanen H., Heliövaara M., Reunanen A. (2002). Flavonoid intake and risk of chronic diseases. Am. J. Clin. Nutr..

[66] Adebamowo C.A., Cho E., Sampson L., Katan M.B., Spiegelman D., Willett W.C. (2005). Dietary flavonols and flavonol-rich foods intake and the risk of breast cancer. Int. J. Cancer..

[67] Arts I.C.W., Jacobs D.R., Gross M., Harnack L.J., Folsom A.R. (2002). Dietary catechins and cancer incidence among postmenopausal women: the Iowa Women’s Health Study (United States). Cancer Causes Control.

[68] Wang L., Lee I.-M., Zhang S.M., Blumberg J.B., Buring J.E., Sesso H.D. (2009). Dietary intake of selected flavonols, flavones, and flavonoid-rich foods and risk of cancer in middle-aged and older women. Am. J. Clin. Nutr..

[69] Wang Y., Gapstur S.M., Gaudet M.M., Peterson J.J., Dwyer J.T., McCullough M.L. (2014). Evidence for an association of dietary flavonoid intake with breast cancer risk by estrogen receptor status is limited. J. Nutr..

[70] Knekt P., Järvinen R., Seppänen R., Hellövaara M., Teppo L., Pukkala E. (1997). Dietary flavonoids and the risk of lung cancer and other malignant neoplasms. Am. J. Epidemiol..

[71] Touvier M., Druesne-Pecollo N., Kesse-Guyot E., Andreeva V.A., Fezeu L., Galan P. (2013). Dual association between polyphenol intake and breast cancer risk according to alcohol consumption level: a prospective cohort study. Breast Cancer Res. Treat..

