# Supplementary material for: Dietary polyphenols and risk of breast cancer in a predominantly low-income population: a prospective analysis in the Southern Community Cohort Study (SCCS)
Source: Am J Clin Nutr. 2025 Mar 22;121(6):1335–45. doi: 10.1016/j.ajcnut.2025.03.017 (PMC12226747; doi:10.1016/j.ajcnut.2025.03.017)
Supplement: multimedia component 1 [file mmc1.docx]

Supplemental Materials

Title: Dietary Polyphenols and Risk of Breast Cancer in a Predominantly Low-Income Population: A Prospective Analysis in the Southern Community Cohort Study (SCCS)

Authors: Lei Fan^#^, Landon T. Fike^#^, Heather Munro, Danxia Yu, Hongwei Si, Martha J. Shrubsole*, Qi Dai*

^#^Co-first author

*Corresponding author

| Supplemental Table S1. Median energy-adjusted intakes of polyphenol classes and subclasses in the Southern Community Cohort Study. | |
| --- | --- |
| Polyphenol, median (25^th^-75^th^ percentile), mg/day | Female |
| Total Polyphenols | 561 (326 - 925) |
| Flavonoids | 239 (141 - 412) |
| Flavanols | 172 (96 - 321) |
| Proanthocyanidins | 95 (60 - 145) |
| Flavanol Monomers | 22 (11 - 57) |
| Flavanol Derivatives | 20 (2.6 - 124) |
| Flavonols | 19 (13 - 27) |
| Flavanones | 12 (3.4 - 37) |
| Anthocyanins | 11 (5.4 - 21) |
| Flavones | 1.3 (0.85 - 1.8) |
| Dihydrochalcones | 0.70 (0.22 - 2.2) |
| Isoflavonoids | 0.32 (0.23 - 0.46) |
| Dihydroflavonols | 0.02 (-0.01 - 0.05) |
| Chalcones | 0.0009 (-0.001 - 0.003) |
| Phenolic Acids | 193 (92 - 455) |
| Hydroxycinnamic Acids | 136 (58 - 408) |
| Hydroxybenzoic Acids | 31 (15 - 53) |
| Hydroxyphenylacetic Acids | 0.36 (0.20 - 0.67) |
| Hydroxyphenylpropanoic Acids | 0.02 (0.01 - 0.05) |
| Lignans | 0.64 (0.45 - 0.92) |
| Stilbenes | 0.04 (0.01 - 0.09) |
| Other Polyphenols | 17 (8.6 - 40) |
| Alkylphenols | 8.6 (3.5 - 23) |
| Alkylmethoxyphenols | 1.4 (0.35 - 4.5) |
| Tyrosols | 1.0 (0.64 - 1.8) |
| Methoxyphenols | 0.12 (0.008 - 0.60) |
| Furanocoumarins | 0.11 (0.07 - 0.16) |
| Phenolic_terpenes | 0.09 (0.04 - 0.25) |
| Hydroxycoumarins | 0.05 (-0.04 - 0.12) |
| Hydroxybenzaldehydes | 0.04 (-0.0006 - 0.07) |
| Hydroxyphenylpropenes | 0.04 (0.02 - 0.06) |
| Naphtoquinones | 0.03 (0.01 - 0.09) |
| Curcuminoids | 0.02 (0.009 - 0.06) |
| Hydroxybenzoketones | 0.002 (-0.003 - 0.006) |
| Other Polyphenols | 0.30 (0.08 - 3.0) |

| Supplemental Table S2. Stratified analysis by human epidermal growth factor receptor 2 (HER2) status | | | | | | | | | | | |
| --- | --- | --- | --- | --- | --- | --- | --- | --- | --- | --- | --- |
| Polyphenol | Quintile 1 (lowest) | | Quintile 2 | | Quintile 3 | | Quintile 4 | | Quintile 5 (highest) | | *P*_-trend_ |
|  | HR | Cases | HR (95%CI) | Cases | HR (95%CI) | Cases | HR (95%CI) | Cases | HR (95%CI) | Cases |  |
| HER2 + |  |  |  |  |  |  |  |  |  |  |  |
| Total Polyphenols | 1.00 (ref) | 26 | 1.25 (0.73 - 2.16) | 32 | 0.92 (0.51 - 1.65) | 26 | 1.07 (0.60 - 1.92) | 29 | 1.18 (0.64 – 2.19) | 28 | 0.7553 |
| Flavonoids | 1.00 (ref) | 23 | 1.51 (0.88 - 2.60) | 37 | 0.84 (0.45 - 1.57) | 22 | 1.00 (0.54 - 1.84) | 27 | 1.28 (0.70 - 2.33) | 32 | 0.6892 |
| Flavones | 1.00 (ref) | 21 | 1.32 (0.73 - 2.37) | 28 | 1.18 (0.64 - 2.17) | 28 | 1.37 (0.74 - 2.53) | 31 | 1.26 (0.66 – 2.43) | 33 | 0.6301 |
| Phenolic Acids | 1.00 (ref) | 28 | 0.89 (0.51 - 1.56) | 27 | 0.97 (0.56 - 1.68) | 32 | 0.97 (0.56 - 1.67) | 28 | 0.95 (0.52 – 1.74) | 26 | 0.9963 |
| Lignans | 1.00 (ref) | 29 | 0.84 (0.49 - 1.45) | 28 | 0.49 (0.25 – 0.91) | 16 | 1.06 (0.59 - 1.91) | 36 | 0.93 (0.47 - 1.84) | 32 | 0.6651 |
| Stilbenes | 1.00 (ref) | 27 | 0.93 (0.52 - 1.66) | 31 | 0.52 (0.26 - 1.04) | 19 | 0.57 (0.29 - 1.13) | 21 | 1.10 (0.59 – 2.06) | 43 | 0.1650 |
| Other Polyphenols |  |  |  |  |  |  |  |  |  |  |  |
| Alkylphenols | 1.00 (ref) | 27 | 0.66 (0.36 - 1.21) | 19 | 1.11 (0.65 - 1.90) | 33 | 1.16 (0.67 – 2.00) | 34 | 0.91 (0.50 - 1.67) | 28 | 0.8565 |
| Tyrosols | 1.00 (ref) | 34 | 0.52 (0.29 – 0.92) | 20 | 0.76 (0.45 - 1.29) | 29 | 0.66 (0.38 - 1.15) | 25 | 0.89 (0.50 - 1.58) | 33 | 0.6960 |
| HER2 - |  |  |  |  |  |  |  |  |  |  |  |
| Total Polyphenols | 1.00 (ref) | 142 | 1.12 (0.88 - 1.42) | 164 | 1.08 (0.85 - 1.37) | 162 | 1.02 (0.80 - 1.31) | 171 | 0.82 (0.62 – 1.08) | 130 | 0.0419 |
| Flavonoids | 1.00 (ref) | 137 | 1.08 (0.85 - 1.37) | 158 | 1.10 (0.86 - 1.41) | 171 | 1.09 (0.85 - 1.40) | 167 | 0.88 (0.68 - 1.15) | 136 | 0.1378 |
| Flavones | 1.00 (ref) | 121 | 1.31 (1.03 - 1.68) | 170 | 1.23 (0.95 - 1.58) | 167 | 1.16 (0.89 - 1.50) | 162 | 0.97 (0.73 - 1.29) | 149 | 0.1931 |
| Phenolic Acids | 1.00 (ref) | 147 | 1.03 (0.81 - 1.31) | 153 | 1.12 (0.89 - 1.43) | 175 | 0.95 (0.74 - 1.21) | 150 | 0.89 (0.69 - 1.16) | 144 | 0.1548 |
| Lignans | 1.00 (ref) | 125 | 1.24 (0.96 - 1.59) | 158 | 1.36 (1.05 - 1.76) | 180 | 1.21 (0.91 - 1.60) | 165 | 1.09 (0.80 - 1.50) | 141 | 0.7993 |
| Stilbenes | 1.00 (ref) | 138 | 0.94 (0.72 - 1.23) | 132 | 1.25 (0.95 - 1.64) | 181 | 1.12 (0.84 - 1.48) | 165 | 1.07 (0.80 - 1.43) | 153 | 0.8489 |
| Other Polyphenols |  |  |  |  |  |  |  |  |  |  |  |
| Alkylphenols | 1.00 (ref) | 136 | 0.97 (0.76 - 1.24) | 135 | 1.10 (0.86 - 1.40) | 162 | 1.14 (0.89 - 1.46) | 168 | 1.07 (0.83 - 1.39) | 168 | 0.7513 |
| Tyrosols | 1.00 (ref) | 155 | 0.92 (0.73 - 1.16) | 149 | 1.01 (0.80 - 1.28) | 155 | 0.91 (0.71 - 1.16) | 144 | 1.13 (0.87 - 1.47) | 166 | 0.2207 |
| Abbreviations: HER2, human epidermal growth factor receptor 2; HR, hazard ratio; 95%CI, 95% confidence interval. | | | | | | | | | | | |

| Supplemental Table S3. Sensitivity analyses for associations between polyphenol intakes and breast cancer risk after excluding follow-up <12 months in the Southern Community Cohort Study | | | | | | | | | | | | |
| --- | --- | --- | --- | --- | --- | --- | --- | --- | --- | --- | --- | --- |
| Polyphenol | Quintile 1 (lowest) | | Quintile 2 | | Quintile 3 | | Quintile 4 | | Quintile 5 (highest) | | *P*_-trend_ |  |
|  | HR | Cases | HR (95%CI) | Cases | HR (95%CI) | Cases | HR (95%CI) | Cases | HR (95%CI) | Cases |  |  |
| Total Polyphenols | 1.00 (ref) | 198 | 1.15 (0.94 - 1.41) | 234 | 0.94 (0.77 - 1.16) | 204 | 0.99 (0.80 - 1.23) | 233 | 0.87 (0.69 - 1.10) | 187 | 0.0669 |  |
| Flavonoids | 1.00 (ref) | 182 | 1.19 (0.97 - 1.46) | 230 | 1.04 (0.84 - 1.29) | 217 | 1.08 (0.87 - 1.34) | 227 | 0.99 (0.79 - 1.23) | 200 | 0.3412 |  |
| Flavanols | 1.00 (ref) | 192 | 1.08 (0.88 - 1.31) | 231 | 0.99 (0.81 - 1.22) | 226 | 0.93 (0.75 - 1.14) | 212 | 0.92 (0.75 - 1.14) | 195 | 0.2258 |  |
| Proanthocyanidins | 1.00 (ref) | 194 | 1.03 (0.84 - 1.27) | 208 | 1.07 (0.87 - 1.31) | 223 | 0.96 (0.78 - 1.19) | 208 | 0.93 (0.74 - 1.17) | 223 | 0.3252 |  |
| Flavonols | 1.00 (ref) | 185 | 1.13 (0.92 - 1.39) | 219 | 1.06 (0.86 - 1.31) | 215 | 1.14 (0.93 - 1.41) | 233 | 1.01 (0.81 - 1.26) | 204 | 0.8257 |  |
| Flavanones | 1.00 (ref) | 207 | 1.00 (0.82 - 1.22) | 213 | 0.91 (0.74 - 1.11) | 197 | 0.83 (0.67 - 1.02) | 199 | 0.92 (0.74 - 1.13) | 240 | 0.3728 |  |
| Anthocyanins | 1.00 (ref) | 175 | 1.12 (0.91 - 1.38) | 214 | 1.13 (0.91 – 1.40) | 225 | 1.07 (0.86 - 1.35) | 218 | 1.03 (0.81 - 1.31) | 224 | 0.6126 |  |
| Flavones | 1.00 (ref) | 161 | 1.34 (1.08 - 1.65) | 225 | 1.24 (1.00 - 1.55) | 222 | 1.17 (0.94 - 1.47) | 222 | 1.13 (0.89 - 1.43) | 226 | 0.7838 |  |
| Phenolic Acids | 1.00 (ref) | 207 | 0.98 (0.80 - 1.19) | 208 | 1.05 (0.86 - 1.29) | 235 | 0.96 (0.78 - 1.17) | 217 | 0.83 (0.66 – 1.04) | 189 | 0.0548 |  |
| Hydroxycinnamic Acids | 1.00 (ref) | 197 | 1.11 (0.90 - 1.36) | 225 | 1.08 (0.88 - 1.33) | 230 | 0.98 (0.80 - 1.20) | 212 | 0.89 (0.71 - 1.12) | 192 | 0.0697 |  |
| Hydroxybenzoic Acids | 1.00 (ref) | 195 | 1.02 (0.83 - 1.25) | 215 | 1.09 (0.89 - 1.33) | 230 | 0.98 (0.80 - 1.21) | 206 | 0.96 (0.78 - 1.19) | 210 | 0.4828 |  |
| Lignans | 1.00 (ref) | 178 | 1.14 (0.92 - 1.40) | 220 | 1.13 (0.91 - 1.41) | 223 | 1.15 (0.91 - 1.45) | 237 | 0.99 (0.76 - 1.30) | 198 | 0.5895 |  |
| Stilbenes | 1.00 (ref) | 191 | 0.90 (0.71 - 1.12) | 190 | 1.02 (0.81 - 1.29) | 230 | 0.98 (0.77 - 1.25) | 223 | 0.99 (0.77 - 1.26) | 222 | 0.8706 |  |
| Other Polyphenols |  |  |  |  |  |  |  |  |  |  |  |  |
| Alkylphenols | 1.00 (ref) | 183 | 0.96 (0.78 - 1.19) | 178 | 1.12 (0.91 - 1.38) | 220 | 1.23 (1.00 - 1.52) | 246 | 1.10 (0.88 - 1.38) | 229 | 0.6033 |  |
| Alkylmethoxyphenols | 1.00 (ref) | 215 | 0.99 (0.82 - 1.21) | 205 | 1.14 (0.94 - 1.38) | 236 | 0.95 (0.77 - 1.16) | 203 | 0.92 (0.74 - 1.14) | 197 | 0.1926 |  |
| Tyrosols | 1.00 (ref) | 211 | 0.90 (0.74 - 1.10) | 202 | 0.93 (0.76 - 1.14) | 198 | 0.96 (0.78 - 1.18) | 202 | 1.22 (0.98 - 1.52) | 243 | 0.0127 |  |
| Models adjusted for age, race, household income, enrollment source, BMI category, physical activity, smoking status, alcohol drinking status, menopausal status, comorbidity index, total energy intake, healthy eating index and family history of breast cancer. *P*_-trend_ obtained by using the medians of quintiles as a continuous variable in the Cox Proportional Hazards models. Abbreviations: HR, hazard ratio; 95%CI, 95% confidence interval. | | | | | | | | | | | | |
